# Transcriptome Analysis Revealed the Molecular Mechanism of Acetic Acid Increasing *Monascus* Pigment Production in *Monascus ruber* CICC41233

**DOI:** 10.3390/jof11010049

**Published:** 2025-01-09

**Authors:** Yan Wang, Weiwei Wu, Xiaoshu Wu, Weiyu Li, Jingjing Cui, Chuannan Long

**Affiliations:** 1 School of Life Sciences, Jiangxi Science and Technology Normal University, Nanchang 330013, China; yypz1003@163.com (Y.W.); w725wei@163.com (W.W.); suzi010134@163.com (X.W.); 18379876567@163.com (W.L.); 2 Jiangxi Key Laboratory of Organic Chemistry, Jiangxi Science and Technology Normal University, Nanchang 330013, China; 3 Analysis and Testing Center, Jiangxi Science and Technology Normal University, Nanchang 330013, China

**Keywords:** *Monascus ruber*, *Monascus* pigments, acetic acid, transcriptome analysis

## Abstract

The addition of acetic acid to *Monascus ruber* cultures is usually used to inhibit the growth of heterotrophic bacteria; however, we found that acetic acid also promotes the growth of *M. ruber* CICC41233, as well as the synthesis of *Monascus* pigments (MPs). Compared with no acetic acid or HCl addition, the diameter of *M. ruber* CICC41233 colonies increased significantly under acetic acid conditions. On the sixth day of fermentation, the yield of total pigments in *M. ruber* increased significantly by 9.97 times (compared with no acetic acid) and 13.9 times (compared with hydrochloric acid). The transcriptomics data showed that the differentially expressed genes between *M. ruber* with acetic acid and without acetic acid were mainly involved in starch and sucrose metabolism, glycolysis/gluconeogenesis, pyruvate metabolism, TCA cycle, and oxidative phosphorylation, and that these differentially expressed genes were not involved in amino acid metabolism. Gene expression analysis showed that the relative expression levels of MP synthesis genes (*MpPKS5*, *MppA*, *MpFasB*, *MppB*, *MppD*, and *MppR2*) were significantly up-regulated under acetic acid conditions. This study clarified the metabolic mechanism of acetic acid promoting the growth of *M. ruber* and the synthesis of MPs, which provided some theoretical guidance for the large-scale production of MPs in the industry in future.

## 1. Introduction

*Monascus ruber* is a small filamentous fungus whose fermentation products have a long history in East Asia and have been used for centuries to produce fermented products such as food colorant and pharmaceutical preparations [1,2]. *M. ruber* can synthesize abundant beneficial secondary metabolites in the process of growth and metabolism, such as *Monascus* pigments (MPs), monacolin k, γ-aminobutyric acid, ergosterol, and enzymes [3,4,5,6].

MPs are a mixture of polyketides. More and more studies have shown that MPs have a variety of biological activities, such as anti-inflammatory, anticancer, anti-hyperlipidemic, antioxidant, and antibacterial properties [7,8,9,10]. Therefore, MPs have a very wide application prospect in food, cosmetics, medicine, and other professional fields. However, the details of the MP synthesis pathway remain controversial. At present, researchers generally believe that one unit of acetyl-CoA and five units of malonyl-CoA are used as precursors to synthesize polyketide chromophores under the action of polyketide synthase (PKS), and that β-keto acids are synthesized from short-chain fatty acids produced by the fatty acid synthesis pathway (FAS). The carboxyl groups of these chromophores are attached to the hydroxyl groups of β-keto acids to form two orange pigments, rubropunctatin and monascorubrin. The orange pigments are reduced to yellow pigments, monascin and ankaflavin. Similarly, the orange pigments are converted into red pigments, rubropunctamine and monascorubramine, by amination reaction with NH_3_ [1,11,12].

MPs are a widely used natural and safe food colorant, but the production of MPs cannot meet the needs of the global market. Improving the yield of MPs has therefore always been an important research topic. In recent years, many studies have improved pigment production by manipulating physical conditions such as temperature, light, and magnetic field [13,14]. Studies on *M. ruber* under different fermentation times and high sugar stress conditions have also been published [15]. The optimization of the nutrient composition of the medium and the addition of exogenous chemicals can not only improve pigment yield, but also help to reduce costs. For example, it has been reported that an effective and low-cost fermentation system using corncob hydrolysate as the fermentation substrate has been established, which can effectively increase the yield of MPs and reduce the synthesis of toxic citrinin [16]. Previous studies have shown that the addition of some acetate to the fermentation medium can improve the yield of MPs [17]. However, the molecular mechanism by which acetic acid promotes pigment biosynthesis in *M. ruber* is still unclear.

Comparative transcriptome analysis (RNA-seq) has been widely used to study the metabolism of *M. ruber* [18]. In this study, an experimental group with the same pH condition was designed to eliminate the interference of acidic environments in MP production. RNA-seq technology was used to analyze the differentially expressed genes (DEGs) of three groups of *M. ruber* CICC41233, and to clarify the regulatory mechanism of acetic acid promoting MP biosynthesis. Through transcriptome analysis and comparison, the results will improve our understanding of the molecular mechanism of MP biosynthesis.

## 2. Materials and Methods

### 2.1. Strain Culture and Observation

*M. ruber* CICC41233 was obtained from the China Industrial Microorganism Strain Preservation and Management Center. In previous reports, *M. ruber* CICC41233 was a good candidate strain for pigment production [19]. Rice flour solid medium (RF) was used as the basic medium for *M. ruber* CICC41233 cultivation. RF consisted of the following reagents in g/L: rice flour (40 mesh sieve), 60 g; NaNO_3_, 2 g; KH_2_PO_4_, 1 g; MgSO_4_·7H_2_O, 2 g; agar powder, 15 g; natural pH. Add 2 mL/L of acetic acid (34.94 mM) or a corresponding amount of HCl with the same pH as the experimental group to the RF. *M. ruber* CICC41233 was cultured in three different RFs at 30 °C. The basic RF was named CK, and the RF with acetic acid and HCl was named TA and TH, respectively.

The growth of *M. ruber* CICC41233 on the RF was observed, and the colony size, morphology, and color were recorded from the 2nd day. Meanwhile, the morphology of *Monascus* strains cultured on three media for 6 days was observed and compared.

### 2.2. Fermentation Methods

As a blank control (CK group), the basic fermentation medium was composed of the following reagents (/L): rice flour (40 mesh sieve), 60 g; NaNO_3_, 3 g; KH_2_PO_4_, 1 g; MgSO_4_·7H_2_O, 0.5 g; natural pH. 2 mL/L of acetic acid (added before use) was added to the basic fermentation medium as the fermentation medium of the acetic acid experimental group (TA group). Based on the basic fermentation medium, an appropriate amount of HCl was added until the pH was the same as that of the TA group, which was used as the fermentation medium of the HCl experimental group (TH group). All media were sterilized at 121 °C for 20 min.

*M. ruber* CICC41233 was cultured in three different RFs at 30 °C for 10 days. The spore suspension was prepared by washing the mycelium of *M. ruber* CICC41233 with 10 mL of sterile water and collected by filtration with filter paper. The final concentration of the spore suspension was about 1 × 10^5^ spores per mL and inoculated into the fermentation medium. 100 mL of fermentation medium was added to a 250 mL Erlenmeyer flask in a rotary shaker at 180 rpm under 30 °C for 6 days.

### 2.3. Pigments and Biomass Determination

We compared the production of extracellular pigments (EPs), intracellular pigments (IPs), and total pigments (TMPs) by *M. ruber* CICC41233 in three fermentation media. Fermentation products were collected on the 2nd and 6th day of fermentation, respectively [19]. The fermentation broth precipitate was dried in an electrothermal blast dryer at 80 °C overnight until the weight was kept constant. Biomass was expressed as dry biomass weight (g)/fermentation broth (25 mL). The optical density (OD) value of the pigment solution was detected at 505 nm using a 722 visible spectrophotometer. The absorption spectrum of the MPs was determined by an ultraviolet spectrophotometer in the range of 300–600 nm.

### 2.4. Transcriptome Sequencing Analysis

The products of 2 and 6 days of fermentation (CK-2, TA-2, TH-2, CK-6, TA-6, and TH-6) were collected and centrifuged at 1000 rpm for 30 min, and the supernatant after centrifugation was removed to obtain the mycelial precipitates, which were then immediately frozen in liquid nitrogen and stored at −80 °C until the samples were submitted to Kidio Biotechnology Corporation (Guangzhou, China) for RNA extraction, cDNA library construction, and RNA-seq analysis. The raw data were processed to remove reads containing adapters, poly-N reads, and low-quality reads to obtain clean reads. Meanwhile, the Q20, Q30, and GC contents of the clean reads were calculated to measure the quality of the sequencing results. In addition, the number of sequences mapped per kilobase transcript per million (FPKM) was used to measure gene expression levels. In gene differential expression analysis, read count was normalized and *p* value was calculated according to the negative binomial distribution model. Finally, multiple hypothesis testing was performed to correct the FDR value (false discovery rate). Genes with FDR < 0.05 and |log2(fold-change)| ≥ 2 were considered differentially expressed. Gene function was annotated using KO (KEGG ortholog database) and GO (gene ontology).

### 2.5. Statistical Analysis

Each experiment was performed at least three times under identical conditions. All data are expressed as mean ± standard deviation (SD), and statistical differences between multiple samples were determined by *t*-test in GraphPad Prism 9 software when appropriate. *p* < 0.05 and *p* < 0.01 were considered statistically significant.

## 3. Results and Discussion

### 3.1. Effect of Acetic Acid on Fungal Growth

**Figure 1:**
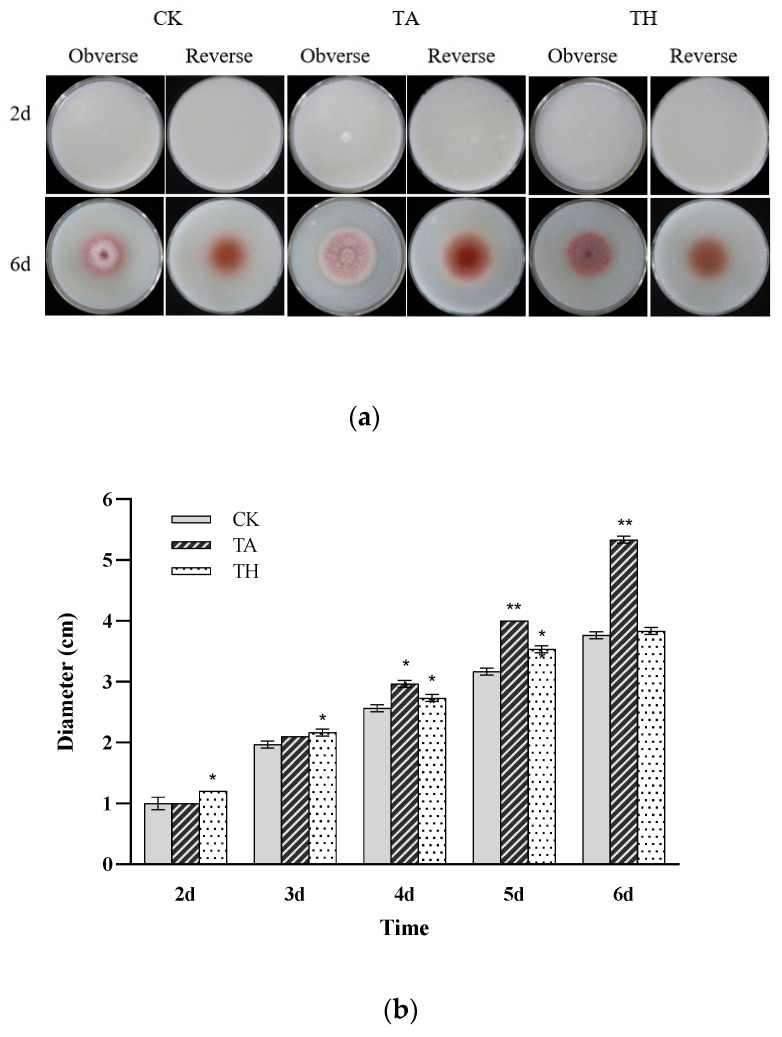
Macroscopic morphology (**a**) and growth diameter (**b**) of *M. ruber* CICC41233 mycelium under different culture conditions. CK, on the fermentation agar plate without addition of acetic acid; TA, on the fermentation agar plate with addition of acetic acid; TH, on the fermentation agar plate with addition of HCl. Each data point is expressed as the mean ± SD (*n* = 3). Statistical significance was considered at *p* < 0.01 (**) and *p* < 0.05 (*) compared with the CK.

The growth of *M. ruber* mycelium played a significant role in the synthesis of secondary metabolites, and the diameter of the mycelium can be utilized as a crucial indicator to reflect the level of pigment biosynthesis [20,21]. In this study, *M. ruber* CICC41233 grew into regular circular colonies on all three media (Figure 1). However, the three groups had significant differences in colony color characteristics. On the sixth day, the CK group and TH group showed a bright red color, while the *M. ruber* mycelium in the TA group was densely distributed and grew further outward, and the diameter of the pigment ring was larger, which showed a dark red color (Figure 1a).

**Figure 2:**
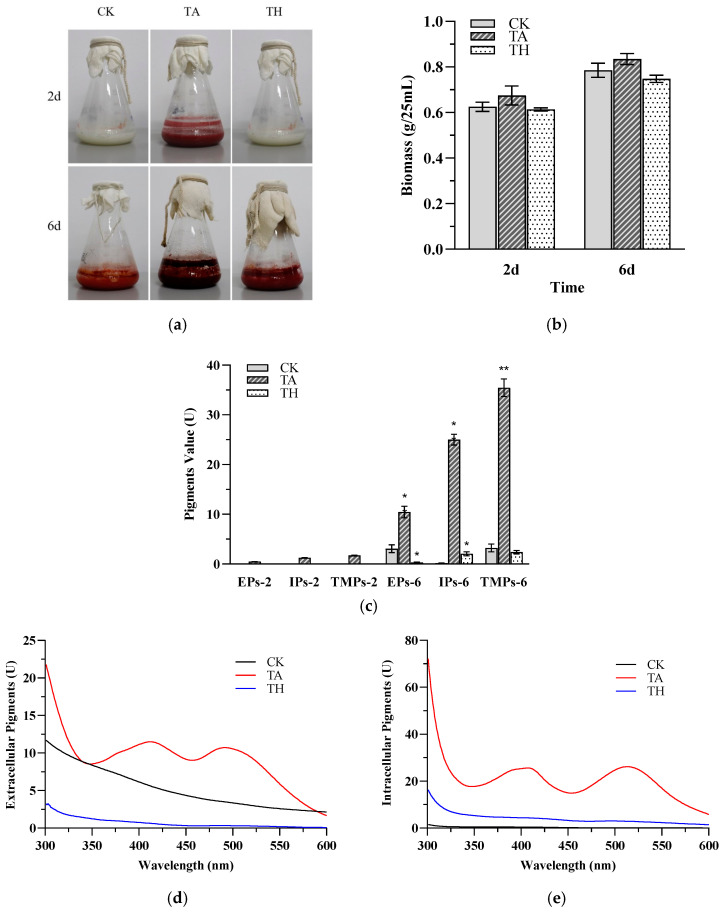
*M. ruber* CICC41233 fermentation and the production of *Monascus* pigments (MPs) under different fermentation conditions. (**a**) Observation of fermentation; (**b**) biomass; (**c**) yield of MPs; (**d**) the extracellular MPs on the sixth day; (**e**) the intracellular MPs on the sixth day. EPs-2, extracellular pigments, 2 days; IPs-2, intracellular pigments, 2 days; TMPs-2, total pigments, 2 days; EPs-6, extracellular pigments, 6 days; IPs-6, intracellular pigments, 6 days; TMPs-6, total pigments, 6 days. Each data point is expressed as the mean ± SD (*n* = 3). Statistical significance was considered at *p* < 0.01 (**) and *p* < 0.05 (*) compared with the CK.

In addition, by measuring the diameter of *M. ruber* CICC41233 on the three media, the growth rate of *M. ruber* in the TA group was faster, and *M. ruber* in the TA group had an obvious growth phenomenon on the second day. The colony diameter on the sixth day was 41.3% larger than that of the CK group and 39.2% larger than that of the TH group (Figure 1b). This was because the short and multi-branched mycelium makes it easier to transport oxygen and nutrients, which promotes the red metabolic pathway and facilitates the synthesis of pigments [22]. In addition, the addition of acetic acid during the fermentation process accelerated the growth of *M. ruber*, so that the total biomass was slightly higher than that of the other two groups (Figure 2b). These results indicated that acetic acid significantly promoted the growth of *M. ruber* mycelium.

### 3.2. Effects of Acetic Acid on the Production of MPs

Many studies have confirmed that *M. ruber* can efficiently produce MPs under acidic conditions, and even inhibit the production of citrinin [23,24,25]. In addition to its function of reducing pH, acetic acid also serves as a regulator of carbon source fermentation. The carbon source provides a carbon skeleton for the growth of *M. ruber*, the precursors of pigment biosynthesis (acetyl-CoA and malonyl-CoA), and energy substances [26,27,28]. On the second day of liquid fermentation, the TA group had significant color characteristics, and began to produce a small number of MPs, while the CK group and TH group did not produce pigment (Figure 2a). On the sixth day of fermentation, the concentrations of EPs, IPs, and TMPs in the TA group were 3.41 times, 148.81 times, and 10.97 times higher than those in the CK group. In contrast, the IPs concentration in the TH group was 12.20 times higher than that in the CK group, and the EPs decreased by 89.51% (Figure 2c). It can be seen that the acidic condition does not play a major regulatory role in the factors that add acetic acid to promote MP production. Moreover, under the same pH conditions, acetic acid further promoted IPs, and also produced a large number of EPs that were easier to extract. In addition, we also observed that the addition of acetic acid during liquid fermentation promoted the accumulation of extracellular yellow pigment (330–450 nm) and intracellular yellow pigment (Figure 2d,e). Similarly, the extracellular pigments produced by *M. purpureus* NRRL 1992 on submerged cultivations with sugarcane bagasse and soy protein isolate also presented two absorption peaks, although at slightly different wavelengths (420–424 and 400–503 nm) [29]. In this sense, it is reported that under the same pH conditions, acetic acid significantly promoted the production of yellow and red pigment in the same soluble MPs (intracellular or extracellular pigment) (Figure 2d,e). These results clearly indicate that the addition of acetic acid can significantly promote the biosynthesis of MPs in the liquid fermentation of *M. ruber*.

### 3.3. Differentially Expressed Genes

RNA-seq was performed to reveal the molecular mechanisms by which acetic acid acts (Tables S1 and S2). The differential gene results showed that there were 1705 DEGs between the CK-2 group and TA-2 group, including 956 up-regulated genes and 749 down-regulated genes (Table 1). There were 263 DEGs between the CK-2 group and TH-2 group, including 115 up-regulated genes and 148 down-regulated genes. There were 3016 DEGs between the CK-6 group and TA-6 group, including 1422 up-regulated genes and 1592 down-regulated genes. There were 1203 DEGs between the CK-6 group and TH-6 group, including 506 up-regulated genes and 697 down-regulated genes. These results showed that the addition of acetic acid promoted the expression level of genes more than that of *M. ruber* CICC41233 under the same pH condition, and that the gene expression level of *M. ruber* also changed at different stages.

### 3.4. Effect of Acetic Acid on the Gene Expression Profile

**Figure 3:**
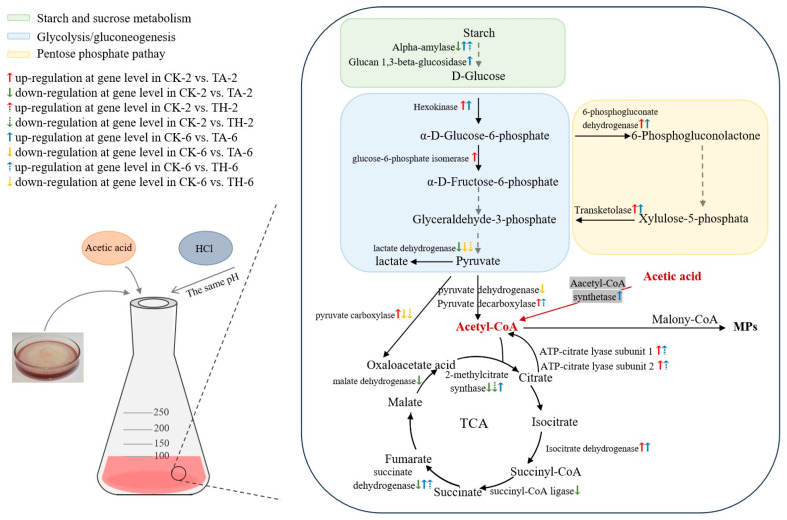
Putative regulatory mechanism behind acetic acid stimulates biosynthesis of *Monascus* pigments.

The KEGG database was used to further analyze the pathways involved in DEGs. Based on the adjusted *p* value (Q value ≤ 0.05), the 20 most significantly enriched KEGG pathways were obtained (Figure S1), as well as the major metabolic pathways involved in DEGs (Table 2, Figure 3).

Metabolic pathway analysis showed that there were some DEGs in the CK-2 vs. TA-2 group involved in carbon metabolism (Table 2). For example, the gene expression levels of glucose-6-phosphate isomerase (evm.TU.Contig1.338), pyruvate carboxylase (evm.TU.Contig10.107), Pyruvate decarboxylase(evm.TU.Contig9.454), 6-phosphogluconate dehydrogenase (evm.TU.Contig2.699), Hexokinase (evm.TU.Contig2.862), fatty-acyl coenzyme A oxidase (evm.TU.Contig3.893), and Transketolase (evm.TU.Contig9.103) were significantly up-regulated (Figure 3, Table S3). In particular, Pyruvate decarboxylase was the key enzyme mediating acetyl-CoA formation. It catalyzed the conversion of glucose into glucose-6-phosphate, which was the first step of glycolysis [30]. Glycolysis was related to the synthesis of acetyl-CoA, so the up-regulation of this gene may increase the occurrence of acetyl-CoA [31]. The 6-phosphogluconate dehydrogenase contributed to the conversion of 6-phosphogluconate into 6-phosphogluconolactone, which provided reducing power for pigment synthesis [32]. Transketolase catalyzed the conversion of xylulose 5-phosphate into glyceraldehyde 3-phosphate, which was the key point of sugar catabolism. These provided sufficient substrates (such as acetyl-CoA and fatty acids) and energy substances (such as ATP, NADH, and NADPH) for cell growth and pigment biosynthesis.

In addition, the addition of acetic acid significantly down-regulated the TCA cycle on the second day of *M. ruber* fermentation (Table 2). Specifically, the genes malate dehydrogenase (evm.TU.Contig12.206), succinate dehydrogenase (evm.TU.Contig2.145), 2-methylcitrate synthase (evm.TU.Contig4.756), and succinyl-CoA ligase (evm.TU.Contig9.301) in the TCA cycle were down-regulated (Figure 3, Table S3). In eukaryotic mitochondria, acetyl-CoA and oxaloacetate undergo a series of oxidative decarboxylations to form NADH, CO_2_, and H_2_O, and finally form oxaloacetate, which entered the cycle with the next acetyl-CoA [33]. The biosynthesis of MPs involved the metabolism of several substances, such as acetyl-CoA, malonyl-CoA, and short- and medium-chain fatty acids. They were precursors of pigment synthesis and had a direct effect on pigment biosynthesis [34,35,36,37]. The overall down-regulation of the TCA cycle reduced the consumption of acetyl-CoA and directed the synthesis of polyketides, while affecting the oxidative phosphorylation pathways and down-regulating the expression of most of the associated DEGs (Table 2). ATP-citrate lyase subunit 1 and ATP-citrate lyase subunit 2 (evm.TU.Contig6.351 and evm.TU.Contig6.350) were the key to catalyzing the formation of acetyl-CoA from citric acid in the cytoplasm, which was up-regulated in the CK-2 vs. TA-2 group (Figure 3, Table S3). Previous studies have shown that the overexpression of ATP-citrate lyase can increase acetyl-CoA and promote starch degradation in rice flour in the fermentation medium, thereby increasing the yield of MPs [19]. This further provided sufficient precursors for pigment synthesis.

The addition of acetic acid had a further effect on the sixth day, and the number of DEGs related to the carbon metabolism pathway increased in the CK-6 vs. TA-6 group (Table 2). Carbohydrate metabolism played an important role in the biosynthesis of MPs, which provided energy and precursors for the growth and metabolism of organisms. In this study, the expression of Alpha-amylase (evm.TU.Contig9.330) and Glucan 1,3-beta-glucosidase (evm.TU.Contig3.414) in the starch- and sucrose-metabolism-related metabolic pathways was up-regulated in the CK-6 vs. TA-6 group (Figure 3, Table S3), which catalyzed the conversion of starch and sucrose into glucose and pyruvate, thereby producing more pigment precursors.

In addition, the addition of acetic acid could significantly up-regulate the transcription levels of some key genes and enzymes, including acetyl-CoA synthetase (evm.TU.Contig6.696) and isocitrate dehydrogenase (evm.TU.Contig6.471) (Figure 3, Table S3). Notably, acetic acid can be converted into acetyl-CoA by acetyl-CoA synthetase, thus providing sufficient precursors for pigment synthesis during the liquid fermentation of *M. ruber* [38,39,40]. Transcriptome analysis showed that it was significantly up-regulated in the CK-6 vs. TA-6 group, while the expression level did not change significantly in the CK-6 vs. TH-6 group. In this way, it can be hypothesized that acetyl-CoA synthetase was a key enzyme indispensable for the promotion of pigment synthesis by acetic acid. Concurrently, enough acetyl-CoA provides more material and energy for pigment synthesis (Figure 3, Table S3). Hexokinase was a key enzyme in glycolysis/gluconeogenesis metabolism. Isocitrate dehydrogenase gene expression was up-regulated to supplement the energy required for pigment synthesis (NADPH and NADH). This indicated that the addition of acetic acid affected the metabolism of carbon sources, and that sufficient energy substrates and pyruvate are further converted into acetyl-CoA [41].

In pyruvate metabolism, the expression of pyruvate carboxylase (evm.TU.Contig10.107) and pyruvate dehydrogenase (evm.TU.Contig9.530) was down-regulated (Figure 3, Table S3). The down-regulation of these genes did not necessarily mean that pyruvate metabolism was inhibited, which may be caused by sufficient starch, nitrogen source, or excessive pyruvate production before fermentation [42]. Accordingly, the gene expression of lactate dehydrogenase (MSTRG.8589) was down-regulated (Figure 3, Table S3), indicating that the conversion of pyruvate into lactic acid was inhibited, and that the reduction in pyruvate consumption could also indirectly prove this conjecture. Pyruvic acid enters the mitochondria to promote its transformation into a precursor of secondary metabolites, which is oxidized to acetyl-CoA and added to the TCA cycle, thereby increasing the production of MPs [43].

However, KEGG analysis of the function of DEGs in the CK-2 vs. TH-2 group and CK-6 vs. TH-6 group showed that the addition of hydrochloric acid did not change many genes in the fermentation of *M. ruber*. Four DEGs in the CK-2 vs. TH-2 group were related to one carbon pool (Table 2). Most of the genes in the CK-6 vs. TH-6 group were enriched in secondary metabolic synthesis pathways, methane metabolism, and nitrogen metabolism (Table 2).

It is worth mentioning that amino acids also play an active role in the synthesis of MPs [44]. For example, “glycine, serine, and threonine metabolism”, “valine, leucine, and isoleucine metabolism”, and “cysteine and methionine metabolism” were involved in the synthesis of MP precursors; many of their metabolites are amine derivatives that constituted red pigments [45]. However, the data suggested that DEGs in the CK-2 vs. TA-2 group and the CK-6 vs. TA-6 group were not involved in amino acid metabolic pathways. These results indicated that the regulatory effect of acetic acid on MP biosynthesis was mainly achieved by affecting the primary metabolic pathway to provide sufficient energy and biosynthetic precursors for secondary metabolism.

In order to determine the dynamic changes in gene expression during the whole fermentation process of *M. ruber*, the transcriptome data from the second and sixth days under the same fermentation conditions were compared (CK-2 vs. CK-6 group, TA-2 vs. TA-6 group, and TH-2 vs. TH-6 group). It is worth noting that compared with the CK-2 vs. CK-6 group and TH-2 vs. TH-6 group, the TA-2 vs. TA-6 group had a large number of DEGs (1362 up-regulated and 1189 down-regulated) (Table S5). During this period, the colony growth of *M. ruber* in the acetic acid environment was the fastest, and the content of MPs continued at a high level, which indicated that the differential genes in the TA-2 vs. TA-6 group were related to the growth and metabolism of *M. ruber*. KEGG analysis showed that 15 pathways were enriched in the TA-2 vs. TA-6 group, mainly distributed in starch and sucrose metabolism, glycolysis/gluconeogenesis, the pentose phosphate pathway, fatty acid degradation, tryptophan metabolism, phenylalanine metabolism, glycine, and serine and threonine metabolism, and so on. The CK-2 vs. CK-6 group enriched six pathways (such as oxidative phosphorylation, glycine, serine, and threonine metabolism, and tyrosine metabolism, etc.), and the TH-2 vs. TH-6 group enriched four pathways (like starch and sucrose metabolism, etc.) (Table S6). These results further indicated that acetic acid had a significant effect on the growth and synthesis of MPs in *M. ruber*.

### 3.5. Acetic Acid Affects the Expression of Pigment Biosynthetic Gene Clusters

**Figure 4:**
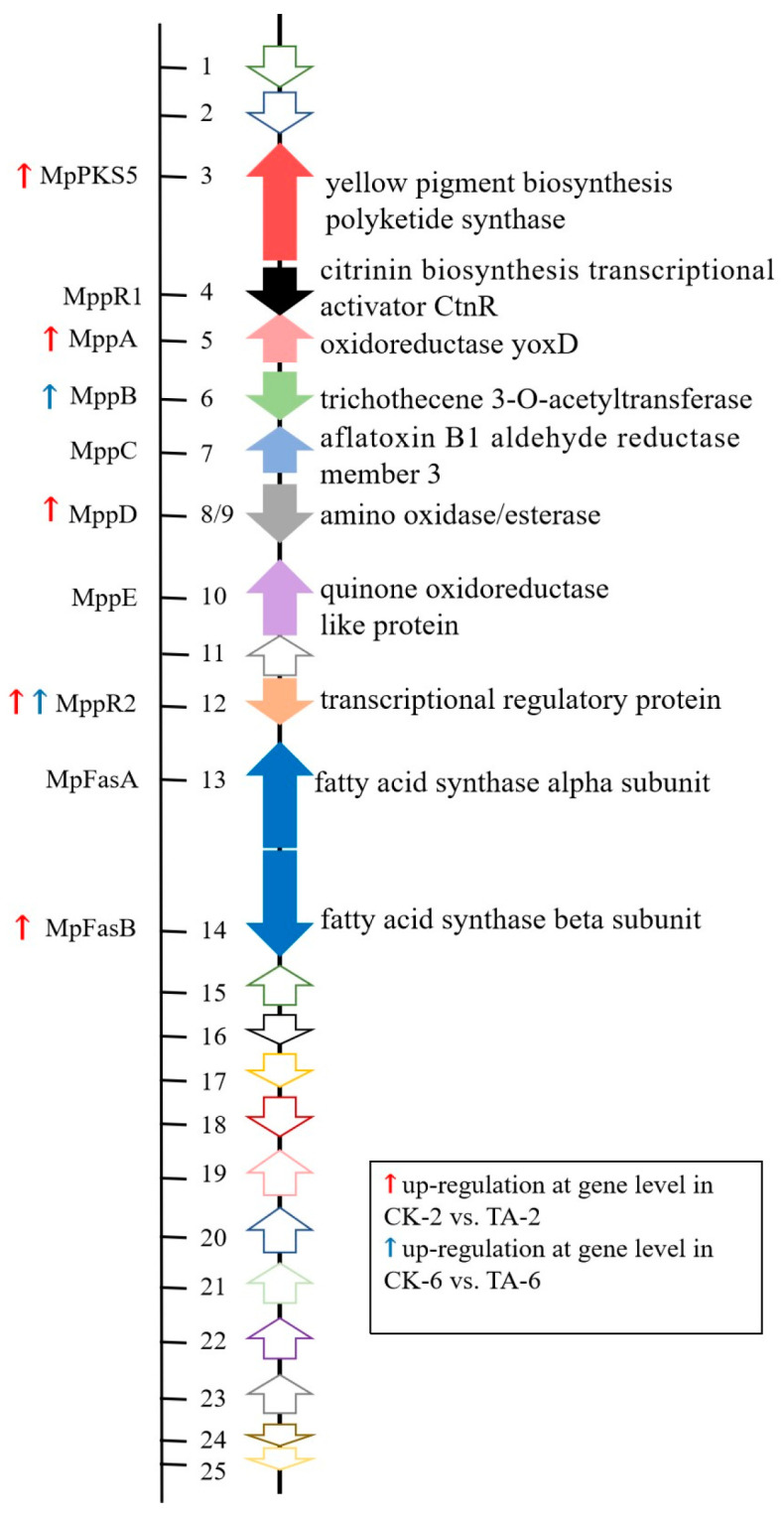
Transcriptional profiles of the *Monascus* pigment synthetic gene cluster. The information on MP biosynthetic gene cluster of *Monascus pilosus* was a reference to obtain MP biosynthetic gene cluster of *M. ruber* CICC41233 (GenBank accession no. KC148521).

The biosynthesis of MPs is regulated by a series of highly conserved gene clusters. The information on the MP biosynthetic gene cluster of *Monascus pilosus* (https://www.ncbi.nlm.nih.gov/nuccore/KC148521.1, accessed on 2 July 2013) was a reference to obtain the MP biosynthetic gene cluster of *M. ruber* CICC41233 (Figure 4, Table S4). The results showed that the key genes *MpPKS5* (*Monascus purpureus* Polyketide Synthase 5, evm.TU.Contig6.566), *MppA* (*Monascus purpureus* pigment A, evm.TU.Contig6.567), *MppD* (*Monascus purpureus* pigment D, evm.TU.Contig6.569), and *MpFasB* (*Monascus purpureus* Fatty acid synthase beta subunit, evm.TU.Contig6.573) involved in pigment synthesis were significantly up-regulated in the TA-2 group (Table 3). The *MppB* (*Monascus purpureus* pigment B, evm.TU.Contig3.926) was significantly up-regulated in the TA-6 group. The *MppR2* (*Monascus purpureus* regulator 2, evm.TU.Contig6.572) was significantly up-regulated in the TA-2 group and TA-6 group, which was a regulatory gene for pigment biosynthesis. The polyketide pathway is considered to be the main biosynthetic pathway of MPs. Acetyl-CoA and malonyl-CoA synthesize polyketide chromophores under the action of polyketide synthase, respectively, and β-keto acids produced by fatty acid synthase. These chromophores and β-keto acids are catalyzed by acyltransferases to form orange pigments [46]. Under the condition of acetic acid (TA-2 group), the activities of polyketide synthase and fatty acid synthase were increased by up-regulating the genes (*MpPKS5*, *MppA*, *MpFasB*, and *MppB*), which promoted the production of chromophores and β-keto acids, thus realizing the esterification and coupling of two branches to synthesize pigments. Under the condition of acetic acid (TA-2 group), the *MppD* was up-regulated, which promoted the conversion of orange pigment into red pigment. It was speculated that the up-regulation of these genes was the reason for the promotion of MP production under acetic acid conditions.

## 4. Conclusions

Based on transcriptomics, this study evaluated the effect of acetic acid on *M. ruber* CICC41233, which provided a theoretical basis for its regulation of MP synthesis. Compared with the same acidic conditions, the addition of acetic acid could promote the growth and pigment production of *M. ruber* CICC41233. The production of EPs, IPs, and TMPs in the TA-6 group was 3.41 times, 148.81 times, and 10.97 times that of the CK-6 group, respectively, and 14.9 times that of the TH-6 group (intracellular and extracellular). Transcriptome sequencing analysis of DEGs showed that these DEGs were mainly focused on primary metabolism (starch and sucrose metabolism, glycolysis/gluconeogenesis, pyruvate metabolism, TCA cycle, and oxidative phosphorylation, etc.), which does not involve the amino acid metabolic pathway. By regulating the expression of key genes in primary metabolism, acetic acid produced sufficient acetyl-CoA and energy (NADH, NADPH, and ATP) and directed them to secondary metabolite synthesis, thereby increasing the production of MPs. Moreover, acetic acid also promotes the expression of genes associated with MPs. In the future, we can utilize genetic engineering techniques to further investigate the potential ways to increase the production of *M. ruber* pigments through the expression of some key genes, and facilitate the large-scale production of MPs in the industry.

## Figures and Tables

**Table 1:** Gene counts of differentially expressed genes (DEGs).

DEG Set	DEG Number	Up-Regulated	Down-Regulated
CK-2 vs. TA-2	1705	956	749
CK-2 vs. TH-2	263	115	148
CK-6 vs. TA-6	3016	1422	1592
CK-6 vs. TH-6	1203	506	697

CK-2, without addition of acetic acid, 2 days; TA-2, with addition of acetic acid, 2 days; TH-2, with addition of HCl, 2 days; CK-6, without addition of acetic acid, 6 days; TA-6, with addition of acetic acid, 6 days; TH-6, with addition of HCl, 6 days.

**Table 2:** KEGG pathway enrichment analysis of DEGs.

DEG Set	Pathway ID	KEGG Pathway	Number of Genes	*p*-Value	Q Value
CK-2 vs. TA-2	ko01100	Metabolic pathways	215	0.000000	0.000000
	ko00190	Oxidative phosphorylation	30	0.000000	0.000000
	ko01110	Biosynthesis of secondary metabolites	94	0.000130	0.004757
	ko01200	Carbon metabolism	32	0.000789	0.021709
	ko00020	Citrate cycle (TCA cycle)	12	0.001445	0.031780
CK-2 vs. TH-2	ko00670	One carbon pool by folate	4	0.000050	0.002296
CK-6 vs. TA-6	ko01100	Metabolic pathways	373	0.000000	0.000008
	ko01200	Carbon metabolism	64	0.000000	0.000016
	ko01110	Biosynthesis of secondary metabolites	183	0.000000	0.000016
	ko00620	Pyruvate metabolism	27	0.000546	0.016528
	ko00010	Glycolysis/Gluconeogenesis	30	0.001118	0.025216
	ko00500	Starch and sucrose metabolism	27	0.001250	0.025216
CK-6 vs. TH-6	ko01100	Metabolic pathways	84	0.000000	0.000000
	ko01110	Biosynthesis of secondary metabolites	60	0.000706	0.020476
	ko00680	Methane metabolism	8	0.000803	0.020476
	ko00910	Nitrogen metabolism	7	0.000803	0.020476

**Table 3:** FPKM (Fragments Per Kilobase Million) value of MP biosynthetic gene cluster in *M. ruber* CICC41233.

NO.	*M. ruber* PKS Cluster	CK-2	TA-2	TH-2	CK-6	TA-6	TH-6	Annotation
1	evm.TU.Contig6.564	1.798	3.513	↑ 5.028	3.552	↑ 10.142	↑ 8.714	d-tyrosyl-tRNA (Tyr) deacylase
2	evm.TU.Contig6.565	57.268	48.2	62.128	40.748	↑ 109.689	53.385	peptidyl-prolyl cis-trans isomerase
3	evm.TU.Contig6.566	1.168	↑ 395.627	1.785	169.533	145.714	↑ 892.257	conidial yellow pigment biosynthesis polyketide synthase
4	evm.TU.Contig1.632	21.037	22.08	19.448	34.911	21.252	26.42	putative citrinin biosynthesis transcriptional activator CtnR
5	evm.TU.Contig6.567	1.854	↑ 242.406	3.301	142.551	79.981	↑ 488.331	putative oxidoreductase yoxD
6	evm.TU.Contig3.926	1.198	0.859	1.605	1.53	↑ 6.306	↑ 6.88	trichothecene 3-O-acetyltransferase
7	evm.TU.Contig9.114	39.896	36.85	39.806	65.793	41.079	41.84	aflatoxin B1 aldehyde reductase member 3
8	evm.TU.Contig6.568	3.202	↑ 2095.71	↑ 8.697	802.36	↓ 301.479	↑ 2611.332	amino oxidase/esterase
9	evm.TU.Contig6.569	0.908	↑ 592.45	2.227	172.647	144.566	↑ 524.957	amino oxidase/esterase
10	evm.TU.Contig8.37	6.594	6.984	5.462	4.655	5.24	↓ 1.723	putative quinone-oxidoreductase-like protein
11	evm.TU.Contig6.571	2.521	↑ 62.562	3.44	45.989	↑ 188.567	↑ 101.542	hypothetical protein
12	evm.TU.Contig6.572	4.083	↑ 144.966	3.779	73.401	↑ 615.452	↑ 225.59	putative transcriptional regulatory protein
13	evm.TU.Contig9.526	124.1	167.777	191.586	289.363	202.502	169.054	fatty acid synthase alpha subunit
14	evm.TU.Contig6.573	0.477	↑ 191.248	0.548	72.198	65.409	↑ 420.957	fatty acid synthase beta subunit
15	evm.TU.Contig5.231	34.316	38.513	33.865	37.819	24.808	24.966	hypothetical protein
16	evm.TU.Contig6.574	3.34	2.49	2.204	1.876	2.269	1.151	GTP-binding protein rhoA
17	evm.TU.Contig1.420	112.352	105.553	129.123	252.165	↓ 34.042	189.907	hypothetical protein
18	evm.TU.Contig6.575	6.612	4.187	6.915	7.51	7.477	7.048	myrosinase-binding protein-like protein
19	evm.TU.Contig6.576	0.194	0.486	0.153	0.772	1.614	1.809	solute carrier family 40 member
20	evm.TU.Contig2.99	5.756	5.317	6.023	9.661	6.999	4.874	hypothetical protein
21	evm.TU.Contig6.578	3.99	6.149	4.076	10.618	9.171	12.568	hypothetical protein
22	evm.TU.Contig6.579	0.165	↑ 299.413	0.13	50.61	↑ 174.723	↑ 274.443	hypothetical protein
23	evm.TU.Contig6.580	0.822	↑ 468.15	1.795	266.275	136.515	↑ 1314.631	salicylate hydroxylase
24	evm.TU.Contig6.581	2.583	↑ 384.686	3.031	173.484	119.056	↑ 631.573	hypothetical protein
25	evm.TU.Contig6.582	1.425	↑ 196.008	1.297	78.137	↑ 499.266	↑ 447.278	putative HC-toxin efflux carrier TOXA

↑, Up-regulated; ↓, Down-regulated. The information on MP biosynthetic gene cluster of *Monascus pilosus* was a reference to obtain MP biosynthetic gene cluster of *M. ruber* CICC41233 (GenBank accession no. KC148521).

## Data Availability

The original contributions presented in this study are included in the article/Supplementary Materials. Further inquiries can be directed to the corresponding authors.

## Supplementary Materials

https://www.mdpi.com/article/10.3390/jof11010049/s1. Table S1, the results of sequence alignment of sample sequencing data and selected reference genomes. Table S2, overview of the quality of *M. ruber* CICC41233 transcriptome data. Table S3, partial genes of *M. ruber* CICC41233 that were changed by acetic acid or HCl. Table S4, information on *Monascus* pigment biosynthetic gene cluster in *M. ruber* CICC41233 based on *M. pilosus*. Table S5, gene counts of Differentially Expressed Genes (DEGs) on different days. Table S6, KEGG pathway enrichment analysis of DEGs on different days. Figure S1, KEGG pathway enrichment of DEGs.
