# Supplementary figures and images for: Transcriptome Analysis Revealed the Molecular Mechanism of Acetic Acid Increasing Monascus Pigment Production in Monascus ruber CICC41233
Source: J Fungi (Basel). 2025 Jan 9;11(1):49. doi: 10.3390/jof11010049 (PMC11767103; doi:10.3390/jof11010049)

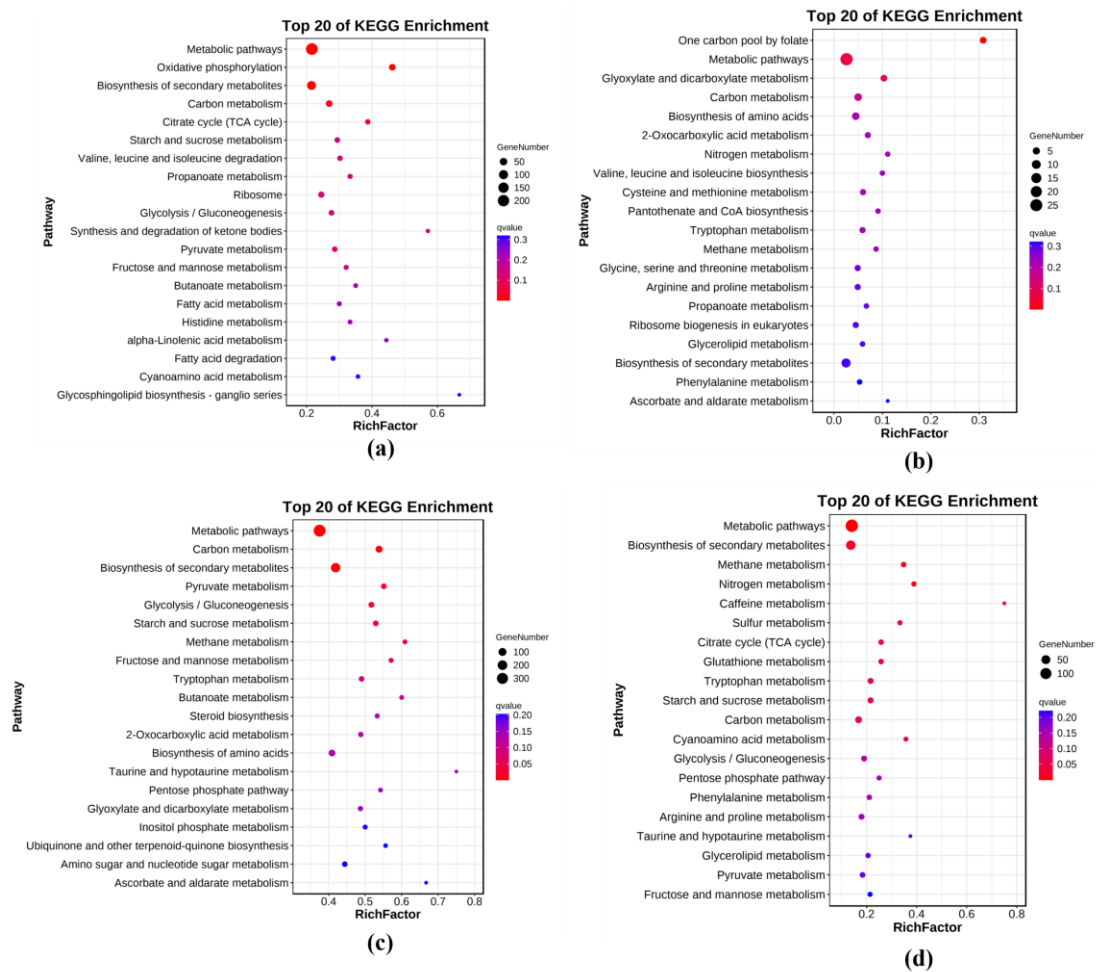

**Figure S1.** KEGG pathway enrichment of DEGs. (a) CK-2 vs. TA-2. (b) CK-2 vs. TH-2. (c) CK-6 vs. TA-6. (d) CK-6 vs. TH-6

Supplement: Supplementary file 1 [file jof-11-00049-s001.zip › Figure S1. KEGG pathway enrichment of DEGs..pdf]
